# Increased Postoperative Bleeding Risk among Patients with Local Flap Surgery under Continued Clopidogrel Therapy

**DOI:** 10.1155/2015/120903

**Published:** 2015-08-06

**Authors:** Wolfgang Eichhorn, Martina Haase, Lan Kluwe, Jürgen Zeuch, Ralf Smeets, Henning Hanken, Manfred Wehrmann, Alexander Gröbe, Max Heiland, Moritz Birkelbach, Carsten Rendenbach

**Affiliations:** ^1^Department of Oral and Maxillofacial Surgery, University Medical Center Hamburg-Eppendorf, Martinistraße 52, 20246 Hamburg, Germany; ^2^Department of Oral and Maxillofacial Surgery, General Hospital Balingen, Tübinger Straße 30, 72336 Balingen, Germany; ^3^Department of Pathology, General Hospital Nürtingen, Auf dem Säer 1, 72622 Nürtingen, Germany

## Abstract

*Purpose*. The purpose of the study was to evaluate the influence of a continued antiplatelet therapy with clopidogrel on postoperative bleeding risk in patients undergoing skin tumor resection and reconstruction with local flaps or skin grafts under outpatient conditions. *Patients and Methods*. The authors designed and implemented a retrospective clinical cohort study at the General Hospital Balingen. The primary endpoint was the bleeding ratio in patients with clopidogrel treatment in comparison to patients without any anticoagulant or antiplatelet therapy. Wound healing was evaluated on days 1, 3, 5, 7, 10, and 14. *Results*. 650 procedures were performed, 123 of them under continued clopidogrel therapy. There were significantly more postoperative bleeding complications among patients with continued antiplatelet therapy. Regarding the whole study population, malignant lesions, a larger defect size, and skin grafts were accompanied by a higher rate of bleeding incidents. However, there were no significant findings in the univariate analysis of the clopidogrel group. All bleeding incidents were easily manageable. *Conclusion*. Despite an increased bleeding ratio among patients under continued clopidogrel therapy, the performance of simple surgical procedures can be recommended. However, cautious preparation and careful hemostasis are indispensable.

## 1. Introduction

There are various indications for the use of clopidogrel as an antiplatelet agent. According to current guidelines, clopidogrel as a single therapy or combined with aspirin is approved for secondary prevention of arteriosclerotic complications in patients with acute coronary syndrome and the necessity of implanting bare metal or drug-eluting stents [1]. Besides, clopidogrel is approved for short-time use following carotid artery intervention and in patients with severe peripheral arterial occlusive disease, and though not clearly indicated, it is even prescribed to patients with atrial fibrillation and an elevated risk of thromboembolic complications [1, 2].

Clopidogrel prevents lifelong clot formation via irreversible inhibition of the platelet P2Y12 adenosine diphosphate receptor while aspirin acts through inactivation of cyclooxygenase [3, 4]. Regarding life capacity of thrombocytes, the anticoagulant effect lasts for 9–11 days [5].

Several studies have shown the decreased number of life-threatening thrombotic complications under single or dual anticoagulant therapy with clopidogrel in these patients [6–9]. Despite the clear indication for drug intake, the question whether or not the antiplatelet therapy should be discontinued during simple surgical procedures as, for instance, minor tumor resections and local flap surgery still is of great relevance and often challenges surgeons and general health care professionals. This fact is mostly due to the paucity of data documenting the occurrence of perioperative bleeding complications [10–13]. Although the latest reports suggest continuation of clopidogrel intake in this regard [14–17], perioperative interruption of antiplatelet medication is still frequently performed due to the fear of an increased risk of severe hemorrhage [18, 19].

The purpose of this study was to evaluate the influence of a continued antiplatelet therapy with clopidogrel on the postoperative bleeding risk in patients undergoing minor tumor resection and reconstruction with local flaps or skin grafts in comparison to patients without pharmaceutical anticoagulation or antiplatelet therapy.

## 2. Materials and Methods

The authors designed and implemented an observational, retrospective single center study including all cases of skin tumor and planned primary or secondary plastic reconstruction via local flap surgery or skin grafts at the General Hospital Balingen, Germany, between June 2005 and March 2015. Patients were excluded from the study population if the surgical procedure was not performed and if postoperative follow-up took place elsewhere and in case of primary bleeding disorder like hemophilia and von Willebrand disease.

The primary endpoint of the study was the bleeding ratio in patients with clopidogrel treatment in comparison to cases without an antiplatelet therapy. Variables with a potential impact on the results were the patients' age and gender as well as localization, size in square millimeters, resection depth, and dignity of the lesion.

Each procedure was considered as an independent case (not the number of the patients) and subsequent analysis was based on this definition.

All surgical operations were elective cases and each intervention was performed by the first author himself under local anesthesia with articaine 4% and ultracain 1 : 200 000 (Ultracain D-S, Sanofi Aventis) under outpatient conditions. If contraindicated, scandicain 4% (Scandicain 4%, AstraZeneca) was infiltrated.

All primary malignant tumors were resected by Mohs procedure and temporarily closed using sterile skin replacements (Epigard, Eschbach, Germany) that were fixed with sutures (Ethilon 5.0, Ethicon, Norderstedt, Germany).

Plastic reconstruction using local flaps or skin grafts was performed primarily within the group of benign lesions and secondarily within the group of malignant lesions following excision and temporary wound closure and after reception of the histological examination results. In case of tumor infiltration of the surgical margin, a follow-up resection was performed prior to plastic reconstruction to secure safe margins.

Local hemostasis was achieved via meticulous bipolar cautery and ligation of all opened vessels. Each primary and secondary wound closure was performed with 5.0 Ethilon sutures (Ethilon 5.0, Ethicon, Norderstedt, Germany). Free skin grafts were stitched and adapted with a sutured compression bandage for 14 days. All other wounds were protected with a standard dressing.

After surgery each patient was observed for 30 minutes before release. Postoperative pain was treated with ibuprofen 400 mg every six hours or beyond if necessary. Postinterventional follow-up was carried out on days 1, 3, 5, 7, 10, and 14. A postoperative bleeding event was defined as an event that required additional treatment.

Data were recorded using Evident (Evident, Bingen, Germany) and SPSS was used for statistical analysis with *t*-test, Chi-square test, and univariate analysis. A *P* < 0.05 was considered significant.

## 3. Results

A total of 650 surgical procedures were performed in 235 patients between June 2005 and March 2015. The control group without an antiplatelet therapy included 527 procedures, whereas 123 surgical interventions were performed under continued clopidogrel therapy. There were 75 patients with clopidogrel monotherapy and 48 patients under dual antiplatelet therapy with clopidogrel and aspirin.

Postoperative bleeding incidents occurred in 1.7% of the cases in the control group (*n* = 9/527) and in 4.9% of all cases in the treatment group (*n* = 6/123). Statistical analysis revealed a significant difference concerning the primary endpoint of this study (*P* = 0.046).

Regarding the appearance of postoperative bleeding events, no statistical difference was found between the groups. In both study groups, the largest number of hemorrhages was recorded within the first 24 hours of follow-up (Table 1).

Among the cases with a postoperative bleeding complication, malignant lesions were diagnosed more frequently than benign lesions (*P* = 0.008) and also the type of surgery (*P* < 0.001), defect size in square millimeters (*P* = 0.04), and procedures in the region of the nose (*P* = 0.015) had a significant impact on the occurrence of hemorrhages. In contrast, the variables age and gender had just as little influence on the occurrence of bleeding complications as resection depth in millimeters as the localization of the lesion. Three out of four bleeding incidents associated with skin graft transplantation occurred in the donor region (Table 2). The bleeding ratio was 5.3% among patients with clopidogrel monotherapy and 4.2% among patients under dual antiplatelet therapy. No statistical difference between monotherapy and dual therapy was recorded.

Regarding the feasibility of surgical intervention in case of postoperative bleeding complications no differences were recorded. Surgical intervention was necessary in one out of 6 cases in the clopidogrel group and in 2 out of 9 cases in the control group. Regarding the whole study population, hospitalization due to bleeding complications was necessary in seven cases for further control and monitoring. Finally, univariate analysis of only the patients in the clopidogrel group revealed no significant differences regarding the variables age, gender, dignity of the lesion, type of surgery, localization of the lesion, and defect size and depth (Table 3). No thrombotic complications were recorded among the patients of both groups.

## 4. Discussion

There are several indications for the use of clopidogrel as an antiplatelet therapy, particularly in cardiologic patients. Several studies revealed the necessity of the continuation of drug intake following cardiac and arterial interventions [1, 7, 14, 15, 20]. However, there is little data on the associated bleeding risk for certain surgical procedures sometimes even causing an interruption of the therapy due to the fear of an increased risk of insatiable bleeding [18–20]. The objective of the present study was to evaluate whether there was an increased frequency of postoperative bleeding incidents among patients under clopidogrel treatment following minor surgical procedures.

According to the results of the current study, there is a significantly increased bleeding ratio among cases with continued clopidogrel therapy undergoing simple surgical procedures. These results are in accordance with earlier studies also indicating a higher bleeding ratio following comparable interventions [14, 15, 20]. However, in a recently published analysis of bleeding complications after oral surgery under continued clopidogrel application, no such correlation has been found [2]. This discrepancy may be due to the relatively high number of cases diagnosed with a malignant skin tumor within the current study population.

On closer consideration of the comparative analysis of cases with and without bleeding events and regarding the absolute numbers of complications under clopidogrel therapy, the increased bleeding ratio primarily seems to be correlated with the variables dignity and type of surgery. While bleeding events were rare following tumor excisions, most of the recorded hemorrhages occurred in case of a malignant lesion and the implementation of local flaps or skin grafts. However, statistical analysis did not reveal a significant difference when analyzing only cases under clopidogrel therapy. However, these findings may also be explained by a relatively small number of cases included in the study. This in turn is mostly due the circumstance that clopidogrel is usually prescribed for a limited period of time when combined with aspirin [1] and thus only few patients undergo one of the included procedures.

Altogether, we recorded no life-threatening bleeding incidents during the study period and the management of all complications was relatively simple with local measures. Despite this fact, delaying the time of a surgical intervention may be worth considering when medically acceptable, especially among patients with dual antiplatelet therapy and planned termination of drug intake due to the loss of indication, for instance, 12 months after the implantation of a drug-eluting stent. According to the results of the current study, this particularly applies to necessary local flaps or skin grafts following the resection of a malignant lesion. Of course, deferring the surgical intervention is not an option, when clopidogrel is used as an alternative for aspirin in case of intolerance and when surgical intervention is urgent.

Either way, because all bleeding incidents in the clopidogrel group were easily manageable without stopping or modifying the antiplatelet therapy and considering the fact that discontinuation of the medication may result in fatal thromboembolic events [2], continuing clopidogrel treatment may therefore generally be recommended among all patients undergoing minor skin tumor resections and reconstruction with local flaps or skin grafts.

## 5. Conclusion

Our results clearly indicate a significantly increased bleeding ratio following minor skin tumor resection and reconstruction with local flaps or skin grafts under continued clopidogrel intake in comparison to patients without antiplatelet or anticoagulant therapy under outpatient conditions. Taking into consideration the fact that an interruption of an antiplatelet therapy with clopidogrel may cause severe thromboembolic complications and that all recorded bleeding incidents were easily manageable, continuation of clopidogrel treatment is recommended, unless a delay of the procedure is medically justifiable. Since all bleeding incidents occurred within four days postoperatively, thorough aftercare and 24-hour on-call availability is recommended during this time period.

## Figures and Tables

**Table 1:** Postoperative bleeding complications in control and clopidogrel therapy groups.

Features and parameters	Anticoagulant therapy		Comparison Control versus clopidogrel
	Control	Clopidogrel	
Number of total cases	527	123	
Demographic features			
Age (years)	73 ± 10	76.0 ± 7.9	*P* = 0.001
Male/female	255/272	101/22	*P* < 0.001

Dignity of tumor			
Benign	256	55	No significant difference (*P* = 0.48)
Malignant	271	68	

Postoperative bleeding			
No bleeding	518	117	*P* = 0.046
Bleeding	9	6	
Bleeding incidence [95% confidential interval]	1.7% [0.9–3.2%]	4.9% [2.3–10.2%]	

Time of bleeding after surgery			
Same day	5	1	Too small sample size for statistic evaluation
Day 1	3	4	
Day 3	1	0	
Day 4	0	1	

**Table 2:** Features of bleeding versus nonbleeding cases regardless of clopidogrel therapy.

Features and parameters	Postoperative bleeding		Comparison No bleeding versus bleeding
	No bleeding	bleeding	
Number of total cases	635	15	
Demographic features			
Age (years)	73.3 ± 9.8	75.4 ± 8.8	No significant difference (*P* = 0.41; *P* = 0.44)
Male/female	346/289	10/5	

Type of the lesion			
Benign/malignant	309/326	2/13	*P* = 0.008

Type of surgery			
Skin graft	38	4 (9.5%)	*P* < 0.001
Local flap	178	8 (4.3%)	
Mohs excision	207	2 (1.0%)	
Excision	212	1 (0.9%)	

Subregion of the lesion			
Nose	120	7 (5.5%)	*P* = 0.015 (nose versus all other regions)
Cheek	187	5 (2.6%)	No significant difference (*P* = 0.06)
Forehead	107	1 (0.9%)	
Ear	64	0	
Others	157	2 (1.3%)	

Defect size			
Area (mm^2^)	198.3 ± 212.25	291.8 ± 190.7	*P* = 0.04
Depth (mm)	4.5 ± 3.5	5.6 ± 3.6	No significant difference (*P* = 0.13)

**Table 3:** Features of bleeding cases versus nonbleeding cases under continued clopidogrel therapy.

Features and parameters	Postoperative bleeding		Comparison No bleeding versus bleeding
	No bleeding	Bleeding	
Number of total cases	117	6	
Demographic features			
Age (years)	75.9 ± 8.0	77.2 ± 5.8	No significant difference (*P* = 0.71/*P* = 1.0)
Male/female	96/21	5/1	

Type of tumor			
Benign/malignant	54/63	1/5	No significant difference (*P* = 0.22)

Type of surgery			
Skin graft	11	1 (8.3%)	*P* = 0.40
Local flap	32	3 (8.5%)	
Mohs excision	43	2 (4.4%)	
Excision	31	0	

Subregion of the lesion			
Nose	23	2 (8.0%)	No significant difference (*P* = 0.85)
Cheek	17	1 (5.6%)	
Forehead	22	1 (4.3%)	
Ear	16	0	
Others	39	2 (4.9%)	

Defect size			
Area (mm^2^)	200.4 ± 203.7	234.3 ± 37.6	No significant difference (*P* = 0.31)
Depth (mm)	4.4 ± 2.5	4.3 ± 3.2	No significant difference (*P* = 0.75)
